# Antimycobacterial and immunomodulatory activities of sorafenib in a preclinical mouse model of TB infection through CD4_+_CD25_low_ and CD8_+_CD25_low_ effector T cells

**DOI:** 10.3389/fimmu.2025.1591026

**Published:** 2025-07-23

**Authors:** Raju S. Rajmani, Avadhesha Surolia

**Affiliations:** ^1^ Molecular Biophysics Unit, Indian Institute of Science, Bangalore, India; ^2^ Dr. Reddy’s Institute of Life Sciences, Hyderabad, India

**Keywords:** *Mycobacterium tuberculosis* (*Mtb*), sorafenib (SRB), immunomodulation, alveolar macrophages (AMs), T cells

## Abstract

Tuberculosis is a communicable disease caused by *Mycobacterium tuberculosis (Mtb).* It is one of the major global public health problems that leads to a high morbidity and mortality rate. Drug resistance in *Mycobacterium tuberculosis* (*Mtb*) is another significant and persistent public health concern. The development of effective TB vaccines and treatments requires a better understanding of the intricate interactions between M. tuberculosis and host immunity. We previously reported that sorafenib (SRB) reduces bacterial growth by allosterically inhibiting ornithine acetyltransferase (MtArgJ), an essential enzyme in the arginine biosynthesis pathway of *Mtb*. Here, we report on the antimicrobial activity of sorafenib in preclinical mouse models of tuberculosis. Sorafenib is a potent drug approved by the Food and Drug Administration (FDA) for treating several types of cancer. The current study is focused on the immunomodulation that SRB induces in the host, specifically the immunological response that is triggered to combat the pathogenicity and survival of the bacteria.Here, we show that SRB significantly sterilizes the bacterial burden in chronic infection animal models of tuberculosis by reducing the number of *Mtb*-susceptible alveolar macrophages (AMs), and that SRB is more effective when combined with rifampicin (RIF). In the current study, we documented a new immune modulatory characteristic of sorafenib that, upon SRB treatment, markedly increased effector T cells (Teff - CD4^+^CD25^low^ and CD8^+^CD25^low^) activity and decreased regulatory T cells, the immunosuppressive T cells (Treg- CD4^+^CD25^high^ and CD8^+^CD25^high^) function. In conclusion, our studies revealed that SRB is beneficial for both boosting an efficient T cell response and lowering the tubercular load.

## Introduction

*Mycobacterium tuberculosis* (*Mtb*) is one of the most prolific and lethal pathogens in human history, still killing around 1.6 million people globally each year (1, 2). More than 500,000 people develop drug-resistant forms of TB each year, and this issue of drug resistance is becoming more and more widespread (3). Furthermore, a six-month course of tuberculosis treatment that includes both first and second lines of treatment with different antibiotics increases the risk of emergence of drug-resistant bacteria (4–7). Additionally, lengthy treatment with a very intense medication regimen and Mtb’s capacity to produce persister bacteria significantly reduces the effectiveness of traditional TB treatment (8–11). According to Boshoff et al. (12), the discovery that *Mtb* has become resistant to all the frontline drugs used to treat tuberculosis highlights the urgent need for novel treatment approaches and drugs. However, a number of FDA-approved repurposed medications are being examined in various clinical trial stages to treat tuberculosis infection and reduce the length of time that patients with tuberculosis must receive treatment (13). Using host-directed therapies that target the pathogen’s intracellular survival holds immense potential for creating anti-tuberculosis (anti-TB) medications. Additionally, this approach may be useful in not only combating drug-resistant strains but also in reducing the likelihood of the emergence of new drug-resistant strains (14–16). The lung is the primary target of *Mycobacterium tuberculosis* that spreads by infecting lung resident macrophages and other immune cells for pathogenicity, propagation, and dissemination (17).

Nevertheless, little is known about the protective immune responses in tuberculosis (18, 19). Several strategies have been developed by *Mtb* to circumvent and inhibit the host immune system (20). Individuals with active tuberculosis disease are unable to develop sufficient cellular immune responses, despite effector CD4^+^ T-cells being one of the most effective components in host containment of *Mtb* (19, 21, 22). However, it was previously stated that to treat tuberculosis, CD4^+^ Th1 cells and CD8^+^ cytolytic lymphocytes must be activated (23). The two main kinds of macrophages that are present in the lungs are tissue-resident alveolar macrophages (AMs) and interstitial macrophages (IMs).AMs, an M2-type population, have anti-inflammatory properties that contribute to the development of an environment that supports *Mtb* growth and replication, while IMs are associated with an immune environment that is less hospitable to microorganisms (16, 24–27). Sorafenib is a potent drug that has been licensed by the FDA to treat many types of cancers (28–31). As we previously reported, ornithine acetyltransferase (MtArgJ), a crucial enzyme in the arginine biosynthesis pathway of *Mtb*, is allosterically inhibited by sorafenib (SRB), which decreases bacterial growth (32). The current study sought to advance our knowledge of SRB-induced host immunomodulation, specifically how SRB stimulates the host’s immune system to combat pathogenicity, bacterial survival, and possible host-pathogen interactions. Here, we demonstrate that by lowering the quantity of *Mtb*-susceptible alveolar macrophages (AMs), SRB dramatically sterilizes the bacterial load in animal models of tuberculosis with persistent infection. Additionally, we demonstrate that SRB works better when paired with rifampicin (RIF). Sorafenib treatment that significantly enhanced Teff (CD4^+^CD25^low^ and CD8^+^CD25^low^) activity and reduced Treg (CD4^+^CD25^high^ and CD8^+^CD25^high^) function constituting a novel immunomodulatory function of this drug in the context of its anti-tubercular function.

## Materials and methods

### Bacterial culture

Middlebrook 7H9 broth media (Difco) supplemented with 10% albumin-dextrose-catalase (ADC) (Becton, Dickinson), 0.4% glycerol, and 0.05% Tween 80 were used to cultivate Mycobacterium tuberculosis (H37Rv). The cultures were then allowed to reach mid-log phase (OD600nm 0.4–0.7) for experiments.

### Ethics and animal experiment

The Institute Animals Ethical Committee (IAEC) and the Institute Biosafety Committee (IBSC) of the Indian Institute of Science, Bangalore reviewed and approved the work plans for the animal experiments. Following the rules established by the Committee for the Purpose of Control and Supervision of Experiments on Animals, or CPCSEA, the experiment was conducted. A sufficient quantity of female BALB/C mice, aged 6 to 8 weeks, were acquired from the Central Animal Facility at IISc, Bangalore. The animals were housed and maintained at the ABSL-3 laboratory at Centre for infectious disease research (CIDR), Indian Institute of Science (IISc), Bangalore, with feed and water ad libitum and 12 h light and dark cycle. The animals were given two weeks to acclimatize to the ABSL-3 facility prior to infection and all experiments were conducted in the ABSL-3 laboratory of IISc. Bangalore.

### Chronic model of infection and drug dosing

For the chronic model of infection, BALB/c mice of 6 to 8 weeks-old (≈20gm) were infected via aerosol through a Madison aerosol generation instrument calibrated to deliver 100 CFU. Mice were housed for 4 weeks to establish chronic infection of *Mtb* in the lungs (4–6 weeks incubation led to 10^5 -10^6 CFU/lung for the chronic infection model establishment). After 4 weeks of post-infection, the mice were randomly grouped (n=10) as: PBS control (H37Rv infected), Sorafenib (SRB) alone treated, Rifampicin (RIF) alone, and sorafenib + Rifampicin (SRB + RIF) in combination and drug treatment started, SRB; 5 doses weekly at 30 mg/kg.b.wt. via I/P route and RIF; 10 mg/kg.b.wt through drinking water. Based on the pharmacodynamic and chemopreventive effects of sorafenib, the 30 mg/kg. body wt of SRB were found to be a suitable dose for chronic sorafenib administration in mice model (33). On the specific time points of 4- and 8-weeks post-treatment, 5 mice from each cohort were humanely sacrificed by cervical dislocation method to assess the bacterial load in their lungs.

### Bacterial CFU estimation

Lung tissues were weighed and then homogenized in 2 ml of sterile 1× phosphate-buffered saline (PBS), serially diluted to 10, 100 and 1000 folds and plated on 7H11 agar plates supplemented with OADC and PANTA (BD-245114) antibiotic mixture (polymyxin B, amphotericin B, nalidixic acid, trimethoprim, and azlocillin). Plates were incubated at 37°C for 3 weeks before colonies were enumerated.

### Histological analysis and evaluation

Following animal sacrifice, lungs were harvested, and 10% neutral buffered formalin was added to the right upper lobe of the lung tissues, which were then kept until they were prepared for histopathological analysis. During tissue processing, the tissues were embedded in paraffin. Sections of the paraffin-embedded and formalin-fixed tissues, each 4 µm thick, were cut onto glass slides and stained with haematoxylin and eosin (H&E) for imaging and histological analysis.

### Granuloma score

Granuloma scoring was done by counting and evaluating each granuloma separately. Using particular pathological markers, we used a scientific approach for rating the development of granulomas (16, 34). Granulomas that had necrosis received a score of 5, those that did not received a necrosis score of 2.5, and those that had fibrosis received a score of 1. To determine the total granuloma scores, these scores were added together (**Table 1**).

**Table 1:** Methodology of the granuloma scores.

	No. of granuloma w/o necrosis X 2.5	No. of granuloma with necrosis X 5	No. of granuloma with fibrosis X 1	Total granuloma score
Example	5X2.5 = 12.5	1x5 = 5	0X1 = 0	17.5

### Histopathology scores

The histopathological evaluation was done by the histopathology scoring system for the lung tissues. We developed a scientific method using modified Mitchison’s scoring system, wherein we considered the number and areas of granulomas, necrosis, alveolar consolidation along with the type of infiltration of immune cells. The histopathology scores were graded as 0-4, (Severe pathology -4; Moderate pathology -3; mild pathology-2; Minor/minimum pathology -1; No pathology -0**).** (7, 16, 35).

### Processing of lungs for immune cell isolation

The lungs were aseptically removed, diced, and digested in a 2% FBS/DMEM solution containing 0.1 mg/ml of Rochelle DNase and 0.2 mg/ml of liberase. Following that, the mixture was mixed and incubated for 30 minutes at 37°C. After that, the pre-digested lung samples were processed using the standard lung dissociation method in a Miltenyi Biotec moderate MACS dissociator. Following processing, the lung samples was filtered through a 70 µm cell strainer. After the filtration samples were centrifuged for five minutes at 4°C at 1600 rpm, the supernatant was disposed of. A freshly prepared RBC lysis buffer was then used to lyse the RBC-containing pellet, which was then left to remain at room temperature (RT) for five to ten minutes. Each sample received 10 milliliters of PBS to offset the effects of the lysis buffer. The lung cell suspensions were then treated for five minutes on ice with Fc block (BD Bioscience), after which the cells were counted and stained for cell surface markers with a cocktail of antibodies and incubated in the dark at room temperature for 30–45 minutes. Finally, samples were acquired on a BD FACSAria™ Fusion flow cytometer (BD Biosciences, San Jose, CA). The antibodies used in the present investigation are anti mouse CD4-FITC, CD8-PE, CD25-APC, CD11c- PE Cy7, F4/80-APC, and mouse Th1/Th2/Th17 Phenotyping Kit (The BD Biosciences). The BD Biosciences BD FACSDivaTM version 8.0.1 was used to analyze the flow cytometry data. The order of the gating sequence was live (SSC-A+ FSC-A+), FSC-H vs. FSC-A to eliminate doublets (singlet gate), and so on.

### RNA isolation and quantitative real time PCR

The lung tissues were harvested in TRIzol (Sigma-Aldrich), homogenized and incubated with chloroform for phase separation. Total RNA was precipitated from the aqueous layer by treatment with isopropanol. The First Strand cDNA synthesis kit (Applied Biological Materials Inc.) was used to convert an equivalent amount of RNA into cDNA. The cDNA was utilized for quantitative real-time PCR analysis of the relevant genes using SYBR Green (Thermo Fisher Scientific). Gapdh was used as the internal control gene. Primer pairs used for expression analyses are provided below (**Table 2**).

**Table 2:** List of primers used for gene expression analysis.

Genes	Forward primer (5’-3’)	Reverse primer (5’-3’)
Mouse GAPDH	GAGCCAAACGGGTCATCATCT	GAGGGGCCATCCACAGTCTT
Mouse IFN-γ	CACGGCACAGTCATTGAAAG	GCTGATGGCCTGATTGTCTT
Mouse IL-10	GAGCAGGTGAAGAGTGATTT	AGGAGTTGTTTCCGTTAGC
Mouse FoxP3	CCCATCCCCAGGAGTCTT	ACCATGACTAGGGGCACTGTA
Mouse TGF-β	CCCTATATTTGGAGCCTG	GTTGGTTGTAGAGGGCAA

### Statistical analysis

GraphPad Prism software (version 8.0) was used for statistical analysis, and results are presented as mean ± SD. The statistical significance between experimental groups was determined by either one way ANOVA Tukey’s multiple comparison test, *p*-value determined and represented as. ns- non-significant, *p<0.05, **p<0.01, ***p<0.001, ****p<0.0001 or a Student’s t-test was used to determine statistical significance across experimental groups (*P < 0.05, **P < 0.01, ***P < 0.001, ****P < 0.0001, and P > 0.5; n.s. not significant).

## Results

It has previously been demonstrated that sorafenib, an allosteric inhibitor of MtArgJ (Mtb ornithine acetyltransferase), reduces *Mtb* virulence and survival in bacterial cultures and the macrophage infection model (32).This study reports the induction of significant immunomodulation inside the host by sorafenib (SRB) treatment, and SRB markedly reduced the bacterial load in mice chronically infected with *Mtb.* Further, we highlight the advantages of using SRB in conjunction with rifampicin (RIF).

### SRB alone and in combination with Rifampicin treatment significantly reduces the *Mtb* burden in the lungs of *Mtb* infected mice

**Figure 1:**
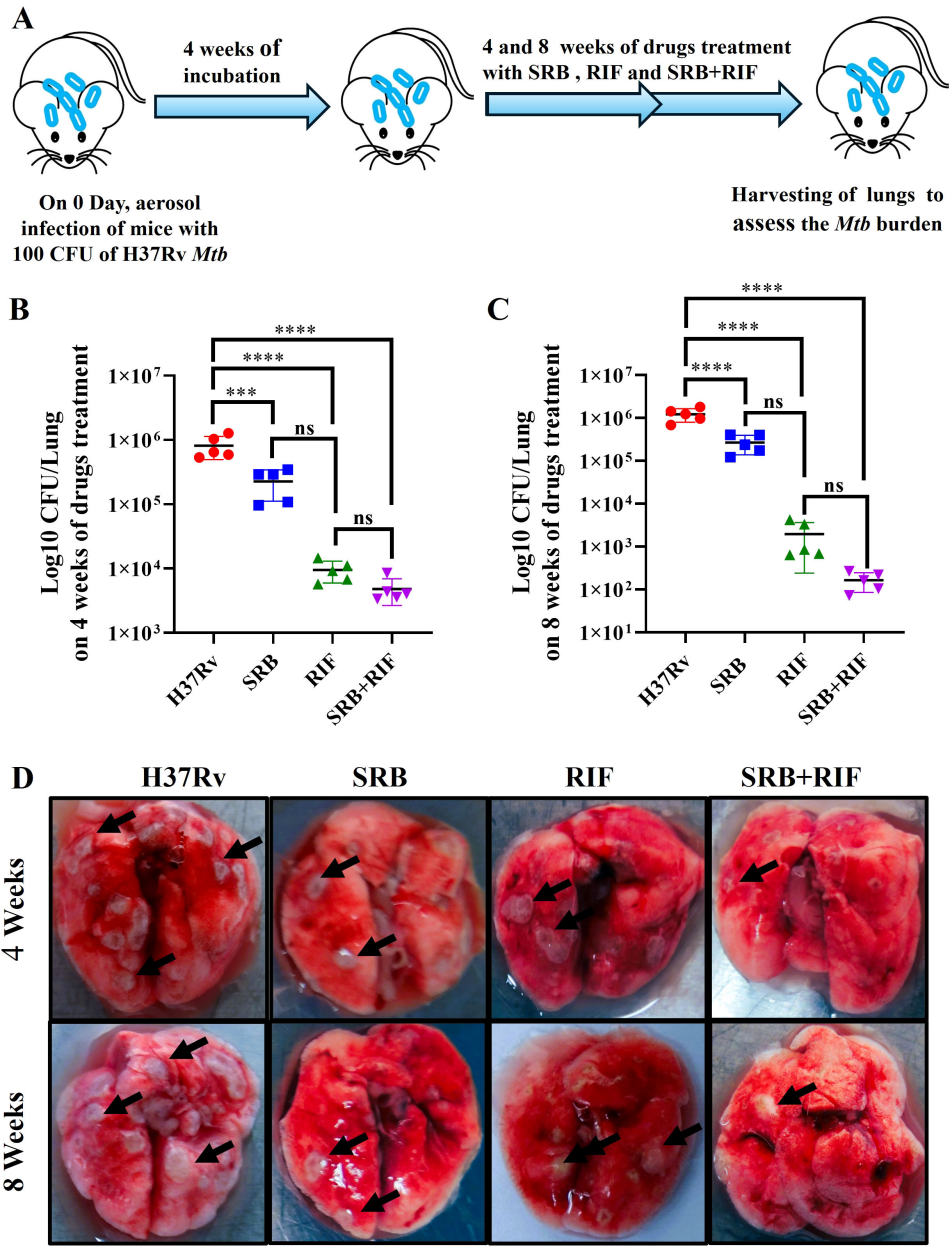
SRB treatment significantly reduces the *Mtb* burden in the lungs of *Mtb*-infected mice. **(A)** Schematic of *Mtb* infection and drug treatment in BALB/C mice. **(B)** Bacterial count in lungs after 4 weeks of post treatment **(C)** Bacterial count in lungs after 8 weeks of post treatment **(D)** Representative gross morphology of lungs of H37Rv infected control, SRB, RIF and SRB+RIF treated (4- and 8-weeks post treatment), the black arrows on lungs correspond to tubercular granuloma.). Data information: the data are collected from 5 mice in each cohort and presented as mean ± SD in log10 CFU/lung and the statistical significance between experimental groups was determined by one way ANOVA Tukey’s multiple comparison test, p-value determined and represented as. ns- non-significant, ***p<0.001, ****p<0.0001.

**Figure 2:**
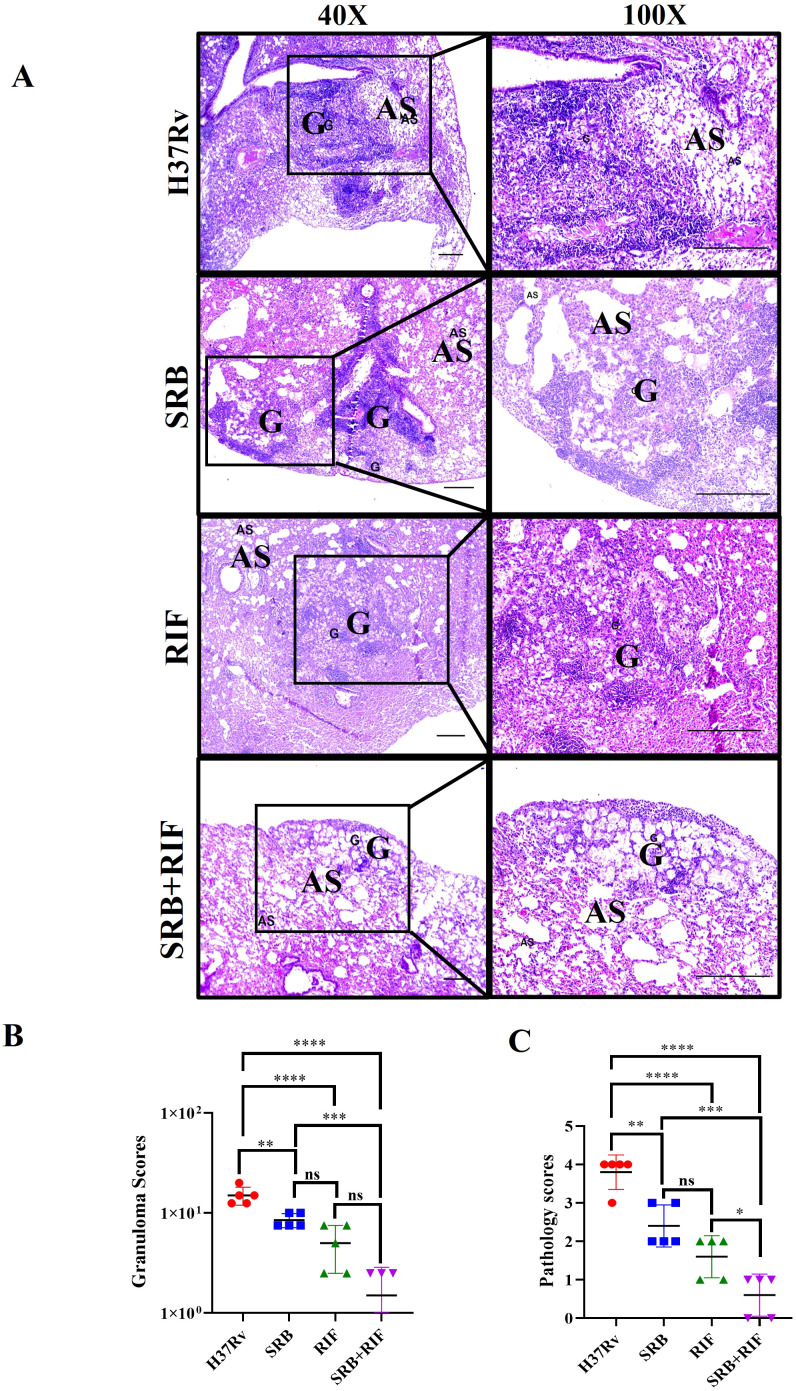
Representative images of histopathology, 8 weeks of post treatment. **(A)** H&E-stained representative images of histopathology of the lungs of the H37Rv infected control, SRB, RIF, and SRB+ RIF treated groups, G-granuloma and AS represent Alveolar space (Scale bars 100um). Histopathological analysis, granuloma score **(B)**, and histopathology score **(C)** of the H37Rv infected control, SRB, RIF, and SRB+ RIF treated groups of chronic infection and treatment models. Data information: data are obtained from 5 biological replicates and presented as mean ± SD; Statistical significance between experimental groups was determined by one way ANOVA Tukey’s multiple comparison test, *p*-value determined and represented as. ns, non-significant, *p<0.05, **p<0.01, ***p<0.001, ****p<0.0001.

In this experiment, we observed the antimycobacterial activity of SRB alone and in combination with RIF. We infected 6 to 8 weeks female Balb/C mice with H37Rv mycobacterial strain through aerosol means (100 CFU), after 4 week of post infection, the mice were randomly grouped as, H37Rv- PBS Control group, Sorafenib (SRB) alone treatment group; Rifampicin (RIF) alone treatment group and sorafenib + Rifampicin (SRB +RIF) in combination treatment group. After 4 and 8 weeks of drug treatments (SRB; 5 doses weekly 30mg/kg.b. wt through I/P route and RIF; 10mg/kg/b.wt. through drinking water daily weekly monitoring revealed no observable signs of toxicity such as weakness, lethargy, or significant weight loss in treated mice. We also observed that the animals in the SRB-treated group were active and healthy throughout the duration of the trial. The mice were humanely sacrificed for accessing bacterial load in lungs (**Figure 1A**) and the small parts of lungs were fixed in 10% neutral buffered formaline for H&E staining. We observed that SRB treatment significantly reduces the *Mtb* burden in the lungs of *Mtb* infected chronic model of infection in mice. Further we noted that SRB in combination with RIF worked better than RIF alone and showed maximum diminution of *Mtb* burden in infected mice in 4 weeks and 8 weeks treatments. The differences in bacterial load between H37Rv control and SRB treated were 0.55 logs in 4 weeks treatment, whereas the differences in bacterial load in the eight weeks treatment between H37Rv control and SRB treated were 0.66 logs in the lungs of mice. Similarly, the bacterial load difference between RIF treated alone and RIF+SRB in combination treated groups was 0.29 logs in 4 weeks treatment, whereas the differences in bacterial load in the 8 weeks treatment between RIF alone and RIF+SRB in combination treated groups was 1.07 logs. (**Figures 1B, C**). The gross pathology of lungs in H37Rv infected control and SRB alone, RIF alone, and RIF+SRB in combination treated groups in 4- and 8-weeks treatment time points, respectively, were found consistent with the CFU count (**Figure 1D**). Severe pathology with multiple small and large granulomas, alveolar consolidation, and infiltration of immune cells in the H37Rv infected control of lungs were observed. Similarly, a moderate to mild pathology in SRB alone and RIF alone treated groups of mice lungs in both 4 and 8-weeks treatment was observed. In RIF+SRB in combination treatment groups, in both 4- and 8-weeks treatment, minimum to no pathology with large areas with normal alveolar space were observed (**Figure 2A**). Granuloma scores and histopathology scores were observed to be consistent with bacterial infection and treatment in H37Rv control, SRB alone RIF alone and SRB+RIF in combination treatment groups respectively (**Figures 2B, C**). The details of granuloma scores are summarized in **Table 1** (**Supplementary Material**; **Supplementary Table 1**). Thus, SRB works better in combination with RIF in reducing the bacterial burden. Collectively, these findings underscore the antimycobacterial efficacy of SRB in chronic TB models

### SRB treatment reduced CD11c^+^ alveolar macrophage/dendritic cells population

**Figure 3:**
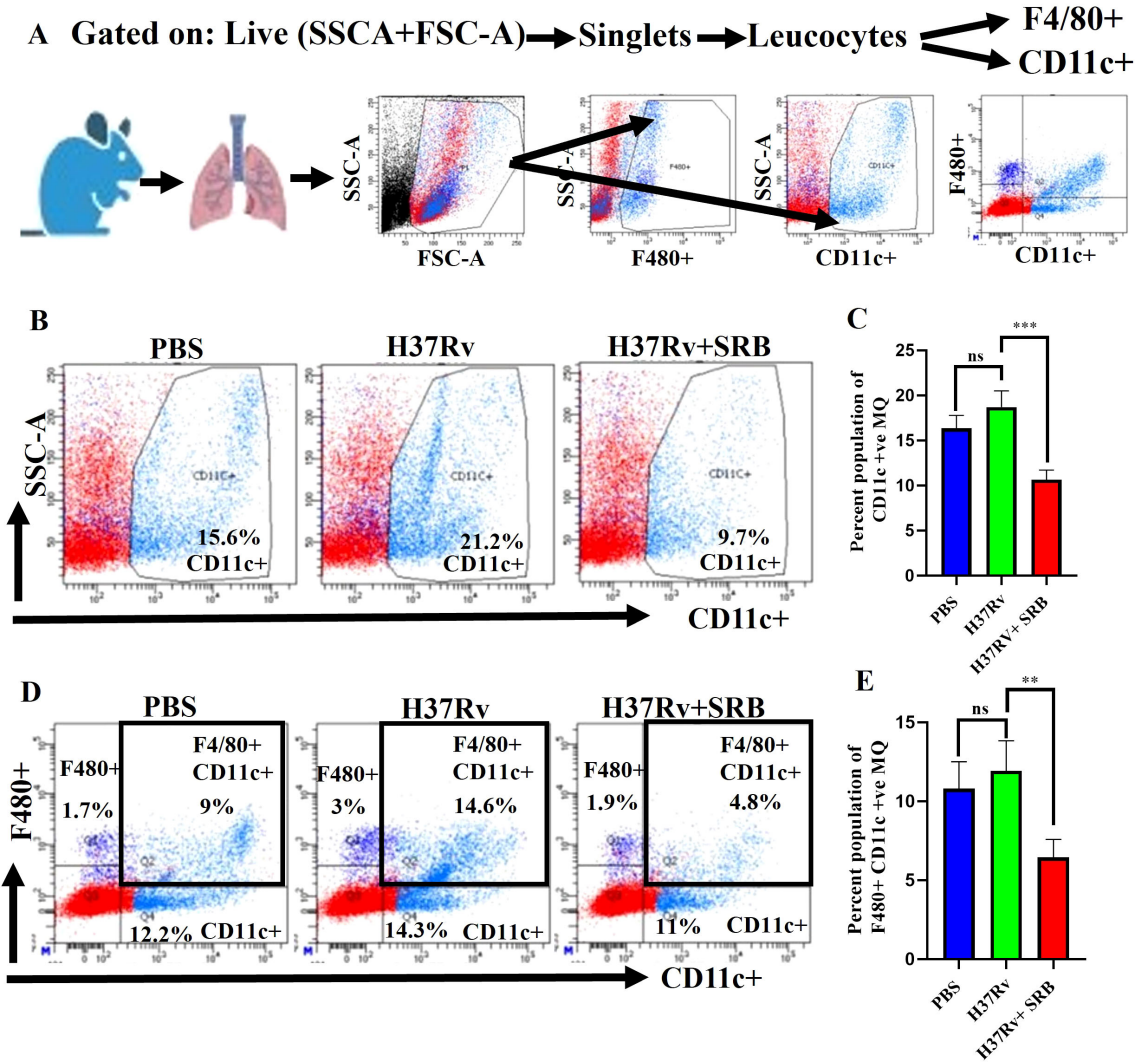
SRB treatment significantly reduced *Mtb* permissive SSC-A+CD11C+ and F4/80+ CD11C+ alveolar macrophages (AMs) population in the chronic model of infection and treatment (8 weeks of post treatment). **(A)** General gating strategy used for Flow cytometry analysis of the lung immune cells obtained from PBS Control, H37Rv infected, and H37Rv+ SRB treated cohorts of mice (n=4). **(B)** Identification of lung alveolar macrophages (AMs; SSC-A+CD11C+) and showing representative FACS dot plot with the percentage populations (% of parent cells acquired) of AMs, **(C)** Bar diagram showing significant reduction of SSC-A+CD11C+ alveolar macrophage population in SRB treated group. **(D)** Identification of lung alveolar macrophages (AMs; F4/80+ CD11C+) showing representative FACS dot plot with the percentage populations (% of parent cells acquired) of AMs, **(E)** Bar diagram showing significant reduction of F4/80+ CD11C+ alveolar macrophage population in SRB treated group). Data information: data are obtained from 4 biological replicates and presented as mean ± SD and the statistical significance between experimental groups was determined by an unpaired Student’s t-test (**P < 0.01, ***p < 0.001, and n.s. not significant).

CD11c is a pan alveolar macrophage (AM)/dendritic cell (DC) marker. There are two primary types of macrophages found in the lung, the tissue-resident alveolar macrophages (AMs; CD11c^+^, Siglec F^+^), and the interstitial macrophages (IMs; CD11b^+^, Ly6C^+,^ and MHC-II^+^), which are derived from monocytes (16, 25, 27, 36, 37). Furthermore, the anti-inflammatory qualities of AMs create an environment that is conducive to *Mtb* replication and spread and IMs promote inflammation and regulate the spread and replication of *Mtb*. (16, 27). In the present study, we found that SRB treated groups had lower populations of double positive, SSC-A^+^CD11c^+^ (**Figures 3A–C**), and F480^+^ CD11c^+^ (**Figures 3D, E**) macrophage phenotypes, which are representative of resident alveolar macrophages (AMs). The decline in the bacterial load in SRB treatment groups is apparently related to a reduction in the alveolar macrophage population. It was reported that monocyte-derived macrophages that express CD11c^high^ are highly susceptible to *Mtb* infection (38). When combined, our results are also in line with previous studies (16, 19).

### SRB treatments up-regulate CD4^+^CD25^low^ CD8^+^CD25^low^ effector T cells

**Figure 4:**
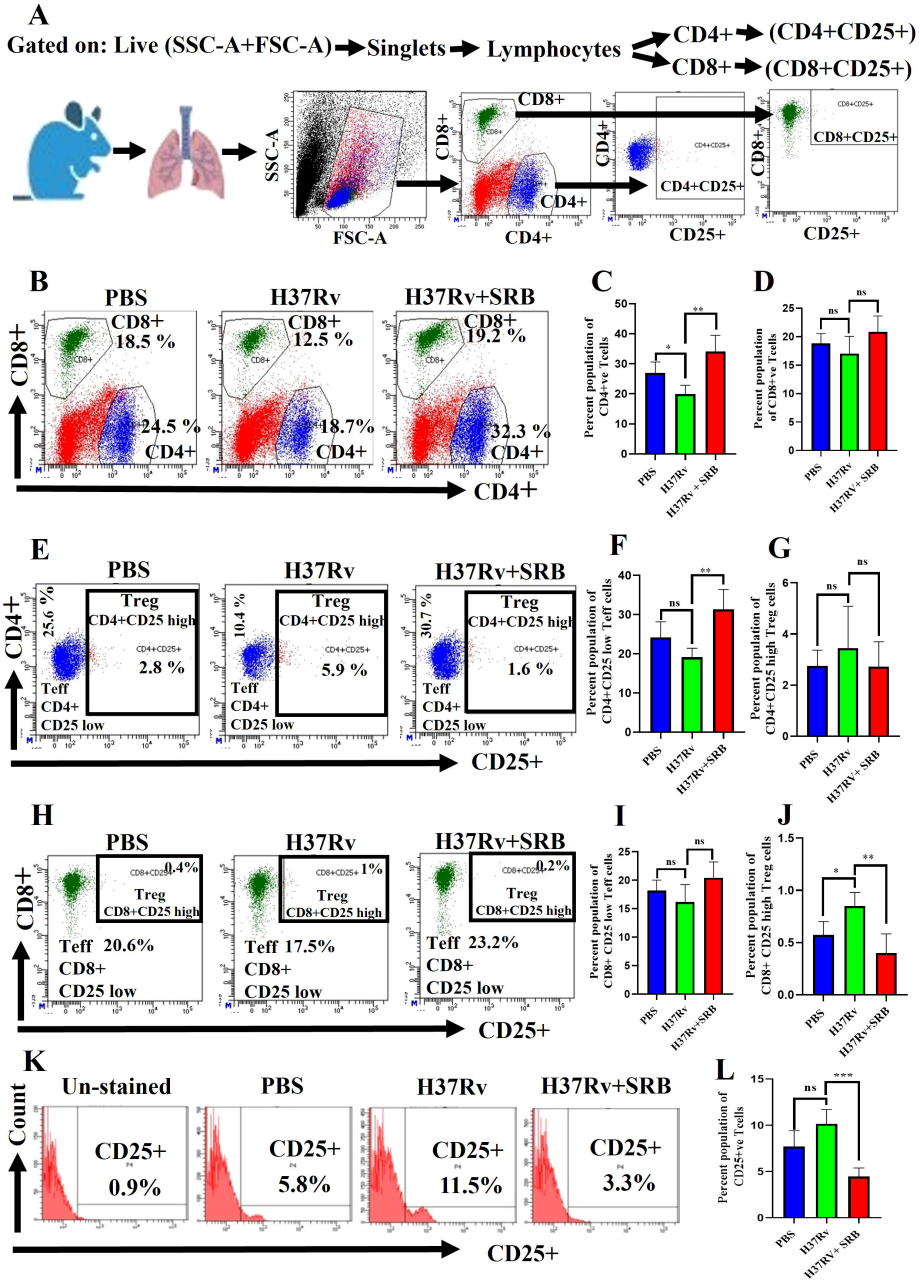
SRB treatment significantly upregulates Teff (CD4^+^CD25^-^ and CD8^+^CD25^-^) in the chronic model of infection and treatment (8 weeks of post treatment). **(A)** General gating strategy used for Flow cytometry analysis of the lung immune cells obtained from PBS Control, H37Rv infected, and H37Rv+ SRB treated cohorts of mice (n=4). **(B)** Identification of lung CD4^+^ and CD8^+^ T cells and showing representative FACS dot plot with the percentage population (% of parent cells acquired) **(C)** Bar diagram showing significant upregulation of CD4^+^ T cells in SRB treated group. **(D)** Bar diagram showing upregulation of CD8^+^ T cells in SRB treated group. **(E)** Identification of lung Teff (CD4^+^CD25^low^) and Treg (CD4^+^CD25^high^) T cells and showing representative FACS dot plot with the percentage population (% of parent cells acquired). **(F)** Bar diagram showing significant upregulation of Teff (CD4^+^CD25^low^) cells in SRB treated group. **(G)** Bar diagram showing non-significant reduction of Treg (CD4^+^CD25^high^) T cells in SRB treated group. **(H)** Identification of lung Teff (CD8^+^CD25^low^) and Treg (CD8^+^CD25^high^) T cells and showing representative FACS dot plot with the percentage population (% of parent cells acquired). **(I)** Bar diagram showing non-significant upregulation of Teff (CD8^+^CD25^low^) T cells in SRB treated group. **(J)** Bar diagram showing significant reduction of Treg (CD8^+^CD25^high^) T cells in SRB treated group. **(K)** Identification of lung CD25^+^ population of T cells and showing representative FACS histograms with the percentage population (% of parent cells acquired). **(L)** Bar diagram showing significant down- regulation of CD25^+^ population T cells in SRB treated group. Data information: data are obtained from 4 biological replicates and presented as mean ± SD and the statistical significance between experimental groups was determined by an unpaired Student’s t-test (*P < 0.05, **P < 0.01, **P < 0.001, ***P < 0.0001, and n.s. not significant).

It is still unclear how the protective immune responses in tuberculosis work (18, 19). However, human tuberculosis control requires the activation of CD4^+^ Th1 cells and CD8^+^ cytolytic lymphocytes (23). Furthermore, by blocking the advantageous antitumor immunity, a subpopulation of suppressive T cells called CD4^+^CD25^+^ regulatory T cells (Tregs) add to the immune suppression seen in hepatocellular carcinomas (HCCs) patients (39–41). Here, we report that, in the chronic model of infection and therapy, SRB-treated mice showed a significant upregulation in CD^+^ T cells population and increase in the CD8^+^ T cell population (**Figures 4A–D**). Further, this study identifies a novel immunomodulatory role for sorafenib in modulating T cell responses during TB infection, which decreased Treg (CD4^+^CD25^high^ and CD8^+^CD25^high^) function (**Figures 4E, G, H, J**) and significantly enhanced Teff (CD4^+^CD25^low^ and CD4^+^CD25^low^) activity (**Figures 4E, F, H, I**) after SRB treatment. Additionally, we noticed that the CD25^high^ T cell population was significantly downregulated in groups that received SRB (**Figures 4K, L**).

### SRB treatments up-regulate Th1^+^ and Th17^+^ CD4^+^T cells and down-regulate FoxP3, IL-10 and TGF-β mRNA expression

**Figure 5:**
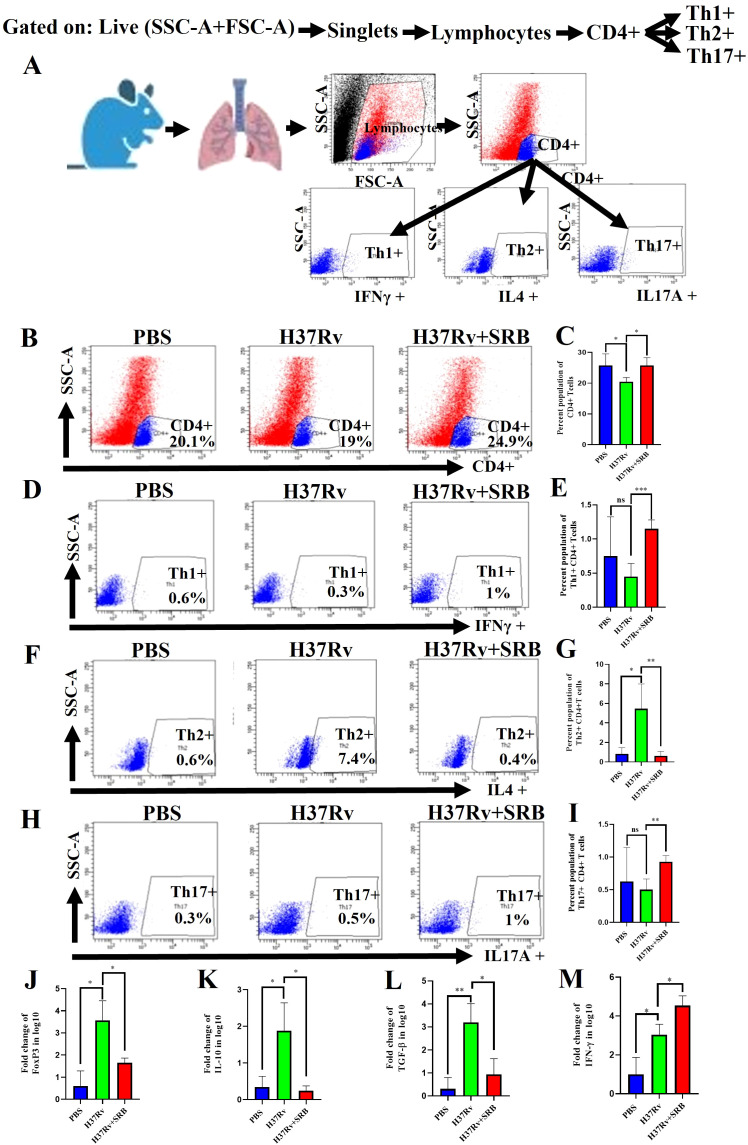
SRB treatments up-regulate Th1^+^ and Th17^+^ CD4^+^T cells and down-regulate FoxP3, IL-10 and TGF-β mRNA expression (8 weeks of post treatment). **(A)** General gating strategy used for Flow cytometry analysis of the lung immune cells obtained from PBS Control, H37Rv infected, and H37Rv+ SRB treated cohorts of mice (n=4). **(B)** Identification of lung CD4^+^ T cells and showing representative FACS dot plot with the percentage population (% of parent cells acquired) **(C)** Bar diagram showing significant upregulation of CD4^+^ T cells in SRB treated group. **(D)** Identification of lung Th1^+^ CD4^+^ T cells and showing representative FACS dot plot with the percentage population (% of parent cells acquired). **(E)** Bar diagram showing significant upregulation of Th1^+^ CD4^+^ T cells in SRB treated group. **(F)** Identification of lung Th2^+^ CD4^+^ T cells and showing representative FACS dot plot with the percentage population (% of parent cells acquired). **(G)** Bar diagram showing significant down-regulation of Th2^+^ CD4^+^ T cells in SRB treated group. **(H)** Identification of lung Th17^+^ CD4^+^ T cells and showing representative FACS dot plot with the percentage population (% of parent cells acquired). **(I)** Bar diagram showing significant upregulation of Th17^+^ CD4^+^ T cells in SRB treated group. **(J)** Bar diagram showing significant down-regulation of FoxP3 mRNA level in log10 fold change in SRB treated group. **(K)** Bar diagram showing significant down-regulation of IL-10 mRNA level in log10 fold change in SRB treated group. **(L)** Bar diagram showing significant down-regulation of TGF-β mRNA level in log10 fold change in SRB treated group. **(M)** Bar diagram showing significant up-regulation of IFN-γ mRNA level in log10 fold change in SRB treated group. Data information: data are obtained from 4 biological replicates and presented as mean ± SD and the statistical significance between experimental groups was determined by an unpaired Student’s t-test (*P < 0.05, **P < 0.01, ***P < 0.001, and n.s. not significant).

Through antigen-driven proliferation and differentiation of naïve T cells, CD4+ T cells can differentiate into distinct subsets of helper T cells, including Th1, Th2, Th17, and Treg cells (42). Th1 cells are the primary immune cells responsible for removal of *Mtb* by secreting the pro-inflammatory cytokines TNF-α and IFN-γ (43). On the other hand, Th17 cells primarily fight *Mtb* by strengthening host defense. (44, 45). Our findings are consistent with these observations. Additionally, we report here that SRB-treated mice showed a significant upregulation in CD4^+^ T cells (**Figures 5A–C**), Th1+ (**Figures 5D, E**), Th17+ (**Figures 5H, I**) population and decrease in the Th2+ T cell population (**Figures 5F, G**). Further, In the present investigation we reported significant downregulation of FoxP3, IL-10 and TGF-β mRNA level and upregulation of pro-inflammatory IFN-γ mRNA level, respectively, in SRB treated group (**Figures 5J–M**).

## Discussion

Designing host-directed, immunomodulation-dependent therapies to manage *Mycobacterium tuberculosis* (*Mtb*) requires an understanding of the linkages and interactions that occur between *Mtb* and immune cells during the infection (16). We previously reported on the direct killing impact of sorafenib (SRB) on *Mtb* by the allosteric inhibition of *Mtb* ornithine acetyltransferase, a crucial enzyme in the arginine biosynthesis pathway resulting in the inhibition of arginine production in the bacteria (32) In the current study, we carried out the infection and treatment experiment to examine the activity of SRB in chronic *Mtb* infection models of mice and examined the host immune effectors that lower the bacterial burden and promote host immune T cells response in SRB-treated mice. We found that the *Mtb* burden in the lungs of mice with a chronic *Mtb* infection is considerably decreased by SRB therapy. Additionally, we also observed that SRB with RIF in combination was more effective than RIF alone. Granuloma and histopathology scores aligned with bacterial load reductions across treatment groups, respectively. Furthermore, we found that SRB-treated groups significantly downregulated CD11c^+^ macrophage phenotype corresponding to resident alveolar macrophage (AMs) population. A decline in the alveolar macrophage population may be the cause of the bacterial load reduction in SRB treatment groups since these macrophages are dependent on fatty acid metabolism and can develop into pro-inflammatory foamy macrophages, whose milieu is favorable for *Mtb* growth and dissemination. According to earlier research, permissive monocytes, dendritic cells, and alveolar macrophages make up the immune cell population most susceptible to *Mtb* infection (16, 26, 27, 46, 47). In the present investigation we reported that SRB-treated mice exhibited a considerable elevation of CD4^+^ T cells and an increase in CD8^+^ T cells in the chronic model of infection and therapy. Additionally, we documented a new immune modulatory characteristic of sorafenib that, upon SRB treatment, markedly increased Teff (CD4^+^CD25^low^ and CD8^+^CD25^low^) activity and decreased Treg (CD4^+^CD25^high^ and CD8^+^CD25^high^) function. Furthermore, we noticed a notable decrease in the CD25^high^ T cell population in those treated with SRB. These findings are consistent with earlier reports, in patients with hepatocellular carcinomas (HCCs), where it has also been demonstrated that sub-pharmacologic doses of sorafenib have unique effects on various subsets of T cells, preferentially increasing Teff (CD4^+^CD25^-^) activation and suppressing Treg (CD4^+^CD25^+^) activity (39–41, 48). Nonetheless, the CD4+ T cell population contains a variety of effectors and memory T cell subsets, including Type-1 T helper (Th1), Th2, and Th17 cells, which develop from naive T cells that have been stimulated to proliferate and differentiate by antigen. Previous studies have demonstrated that human tuberculosis results in a downregulation of Th1/Th17 and CD4 T cell populations and an increase in Th2 and regulatory T cell (Treg) responses (49, 50). Although the Th2 CD4 T cell population aids in *Mtb* proliferation, the Th1/Th17 CD4 T cell population is crucial for treating active TB (23, 51, 52) Additionally, proinflammatory cytokines like IFN-γ aid in inducing Th1-mediated protection in TB, while high IL-4 levels aid in the development of a Th2 response that effectively counteracts protective cytokines and results in a loss of TB control (53). Prior studies have shown that individuals with active TB have greater levels of immunosuppressive cytokines such TGF-β and IL-10 as well as FoxP3+Treg cells wherein the FoxP3 mRNA expression is regulated through the secretion of interleukin 10 (IL-10) or transforming growth factor beta (TGF-β) (54, 55). It has been previously reported that peripheral blood mononuclear cells (PBMCs) from patients with active tuberculosis (TB) had significantly higher proportions of CD4+CD25^high^ T cells and FoxP3 mRNA expression levels than cells from healthy controls (56, 57). However, the protective immune responses in tuberculosis are still not fully understood (18, 19). TB bacteria have developed several defense mechanisms to circumvent and weaken the human immune system (20). People with active tuberculosis disease are unable to develop sufficient cellular immune responses, despite effector CD4^+^ T-cells being one of the most effective components in host containment of the *Mtb* (19, 21, 22). However, it has been previously noted that mycobacterial tuberculosis infection must be fought by activating CD4^+^ Th1 cells and CD8^+^ cytolytic lymphocytes (23). Our results are in line with earlier studies showing that the bacterial load can be significantly affected by altering the lung immune cell population (16, 19, 21, 22, 58, 59). There are also some limitations of our studies, here the short-term infection outcomes were assessed (4 and 8 weeks), the long-term effect remains unknown, and the BALB/c mice used represents partially human immune response to TB. Further, a small cohort size has been used which has a limited statistical power. Testing the efficacy of SRB in drug resistant strain in animal model of TB constitutes another limitation of the study. In summary, our study showed that SRB promotes an effective T cell response and reduces the tubercular load. It also has an additive impact when combined with rifampicin (RIF), which bodes well for its induction in anti-tubercular treatments.

## Data Availability

The original contributions presented in the study are included in the article/**Supplementary Material**. Further inquiries can be directed to the corresponding authors.
