# Supplementary material for: Antimycobacterial and immunomodulatory activities of sorafenib in a preclinical mouse model of TB infection through CD4+CD25low and CD8+CD25low effector T cells
Source: Front Immunol. 2025 Jul 23;16:1591026. doi: 10.3389/fimmu.2025.1591026 (PMC12325335; doi:10.3389/fimmu.2025.1591026)
Supplement: Supplementary file 1 [file DataSheet1.pdf]

Antimycobacterial and immunomodulatory activities of sorafenib in mice preclinical model of TB infection through $CD4^{+}CD25^{low}$ and $CD8^{+}CD25^{low}$ effector T cells.

Raju S Rajmani[1*] and Avadhesha Surolia[1,2*]

Affiliations:

[1]Molecular Biophysics Unit, Indian Institute of Science, Bangalore-560012

[2]Dr. Reddy's Institute of Life Sciences, Hyderabad- 500046

*Corresponding authors.

Raju S Rajmani, raju.rajmani@yahoo.in

Avadhesha Surolia, surolia@iisc.ac.in

Molecular Biophysics Unit, Indian Institute of Science, Bangalore (India)

Phone (+91) 80-22937414

Supplementary material; Supplementary table 1; Details of granuloma scores

Groups for Lung tissues histopathology	Granuloma with Necrosis	Granuloma with no Necrosis	Granuloma with Fibrosis	Granuloma Scores	Granuloma Fraction	Pathology Scores
H37Rv-1	0	8	0	20	40	4
H37Rv-2	0	6	0	15	35	4
H37Rv-3	0	6	0	15	45	4
H37Rv-4	0	5	0	12.5	25	3
H37Rv-5	0	5	0	12.5	30	4
SRB-1	0	4	0	10	25	3
SRB-2	0	4	0	10	20	2
SRB-3	0	3	0	7.5	15	2
SRB-4	0	3	0	7.5	20	3
SRB-5	0	3	0	7.5	20	2
RIF-1	0	1	0	2.5	10	1
RIF-1	0	3	0	7.5	20	2
RIF-1	0	1	0	2.5	5	1
RIF-1	0	2	0	5	10	2
RIF-1	0	3	0	7.5	20	2

SRB+RIF-1	0	1	0	2.5	5	1
SRB+RIF-2	0	1	0	2.5	5	1
SRB+RIF-3	0	1	0	2.5	5	1
SRB+RIF-4	0	0	0	0	0	0
SRB+RIF-5	0	0	0	0	0	0
