# Supplementary material for: Antimycobacterial and immunomodulatory activities of sorafenib in a preclinical mouse model of TB infection through CD4+CD25low and CD8+CD25low effector T cells
Source: Front Immunol. 2025 Jul 23;16:1591026. doi: 10.3389/fimmu.2025.1591026 (PMC12325335; doi:10.3389/fimmu.2025.1591026)
Supplement: Supplementary file 2 [file DataSheet2.pdf]

**Antimycobacterial and immunomodulatory activities of sorafenib in mice preclinical model of TB infection through $CD4^{+}CD25^{low}$ and $CD8^{+}CD25^{low}$ effector T cells.**

Raju S Rajmani[1*] and Avadhesha Surolia[1,2*]

**Affiliations:**

[1]Molecular Biophysics Unit, Indian Institute of Science, Bangalore-560012

[2]Dr. Reddy's Institute of Life Sciences, Hyderabad- 500046

*Corresponding authors.
Raju S Rajmani, raju.rajmani@yahoo.in
Avadhesha Surolia, surolia@iisc.ac.in
Molecular Biophysics Unit, Indian Institute of Science, Bangalore (India)
Phone (+91) 80-22937414

**Supplementary materials.**

**Supplementary figure 1**

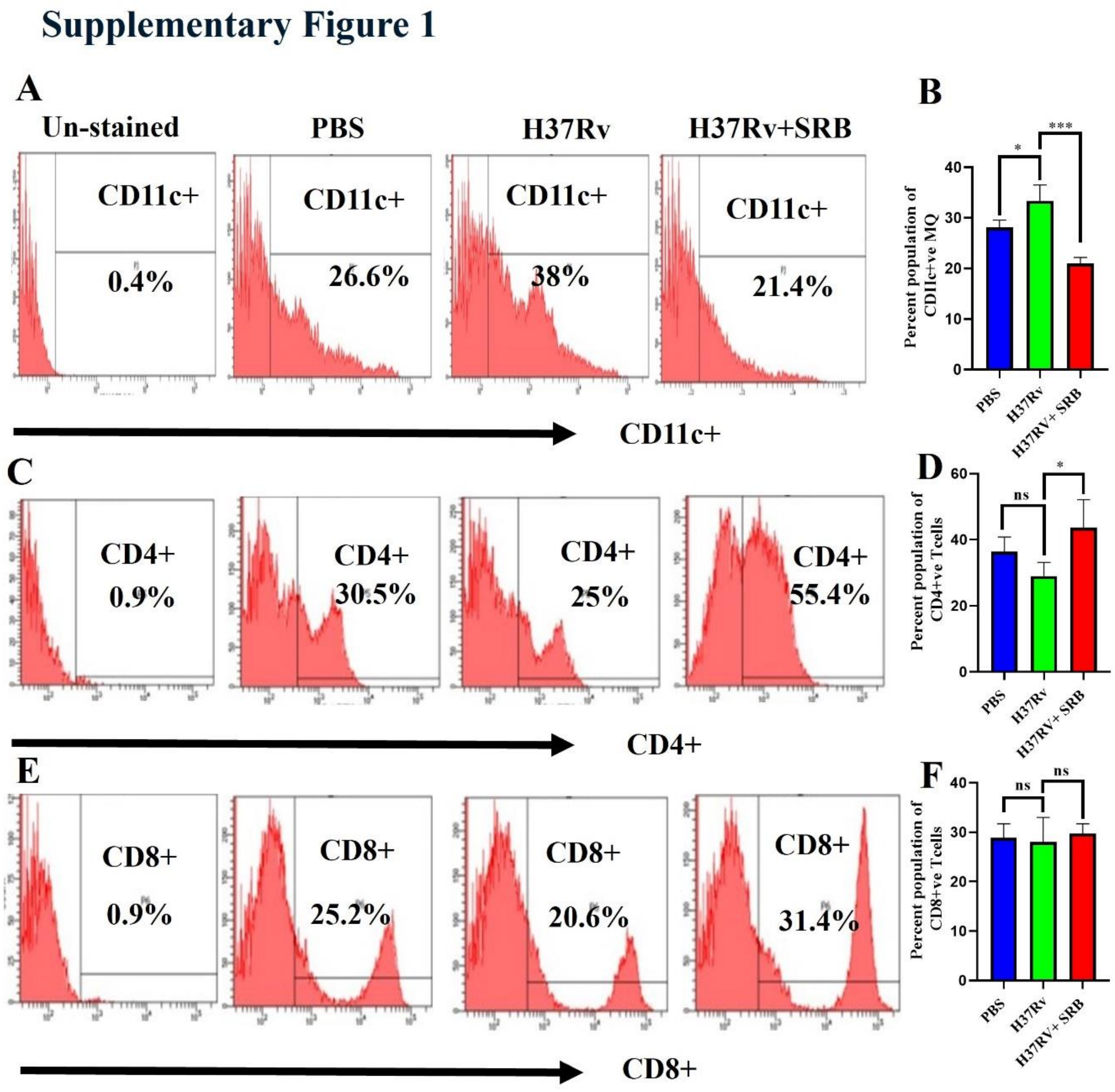


**Supplementary Figure 1:** SRB treatment significantly upregulates $CD4^{+}$ and significantly downregulate CD11c +ve population of macrophage in the chronic model of infection and treatment (8 weeks of post treatment). Flow cytometry analysis of the lung immune cells obtained from Un-stained samples, PBS Control, H37Rv infected, and H37Rv+ SRB treated cohorts of mice (n=4). **(A)** Identification of lung alveolar macrophages (AMs; SSC-$A^{+}CD11C^{+}$) and showing representative FACS histograms with the percentage populations (% of parent cells acquired) of CD11c +ve population of macrophage, **(B)** Bar diagram showing

significant reduction of SSC-$A^+$CD11$C^+$ alveolar macrophage population in SRB treated group. **(C)** Identification of lung $CD4^+$ T cells and showing representative FACS histogram with the percentage population (% of parent cells acquired) **(C)** Bar diagram showing significant upregulation of $CD4^+$ T cells in SRB treated group. **(D)** Identification of lung $CD8^+$ T cells and showing representative FACS histogram with the percentage population (% of parent cells acquired) **(E)** Bar diagram showing non-significant upregulation of $CD8^+$ T cells in SRB treated group. Data information: data are obtained from 4 biological replicates and presented as mean ± SD and the statistical significance between experimental groups was determined by an unpaired Student's t-test (*P < 0.05, **P < 0.01, ***P < 0.001, ****P < 0.0001, and n.s. not significant.

**Sorafenib (SRB) does not exhibit toxicity in mice**

SRB was administered orally five times a week for a maximum of four weeks at a concentration of 30 mg/kg/b/wt to assess the toxic effect of SRB in BALB/c mice. The mice were examined weekly, and no signs of toxicity, including weakness, lethargy, weight loss, skin erythema, or a shaking gait, were observed. Throughout the whole experimental period, all the cohorts of animals remained healthy and alive. There were no appreciable variations in body weights between the PBS and SRB-treated groups **(Fig. 2B).** Blood chemistry analysis was used to measure the levels of serum glutamic pyruvic transaminase (SGPT), serum glutamic-oxaloacetic transaminase (SGOT), creatinine, alkaline phosphatase (ALP), and blood urea nitrogen (BUN). The enzymatic levels of SGPT, SGOT and ALP, which are indicators of liver function, did not significantly change in the SRB-treated group as compared to the PBS-treated groups **(Figs. 2C, 2D &2E).** All animals in the SRB-treated cohorts showed Insignificant alterations in BUN and creatinine levels, which are markers of renal function, when compared to the PBS-treated group **(Figs. 2F & 2G).** These results imply that there were no nephrotoxic effects of SRB. These serum chemistry examinations indicate that SRB is a safe medication for treating tuberculosis.

# Supplementary Figure 2

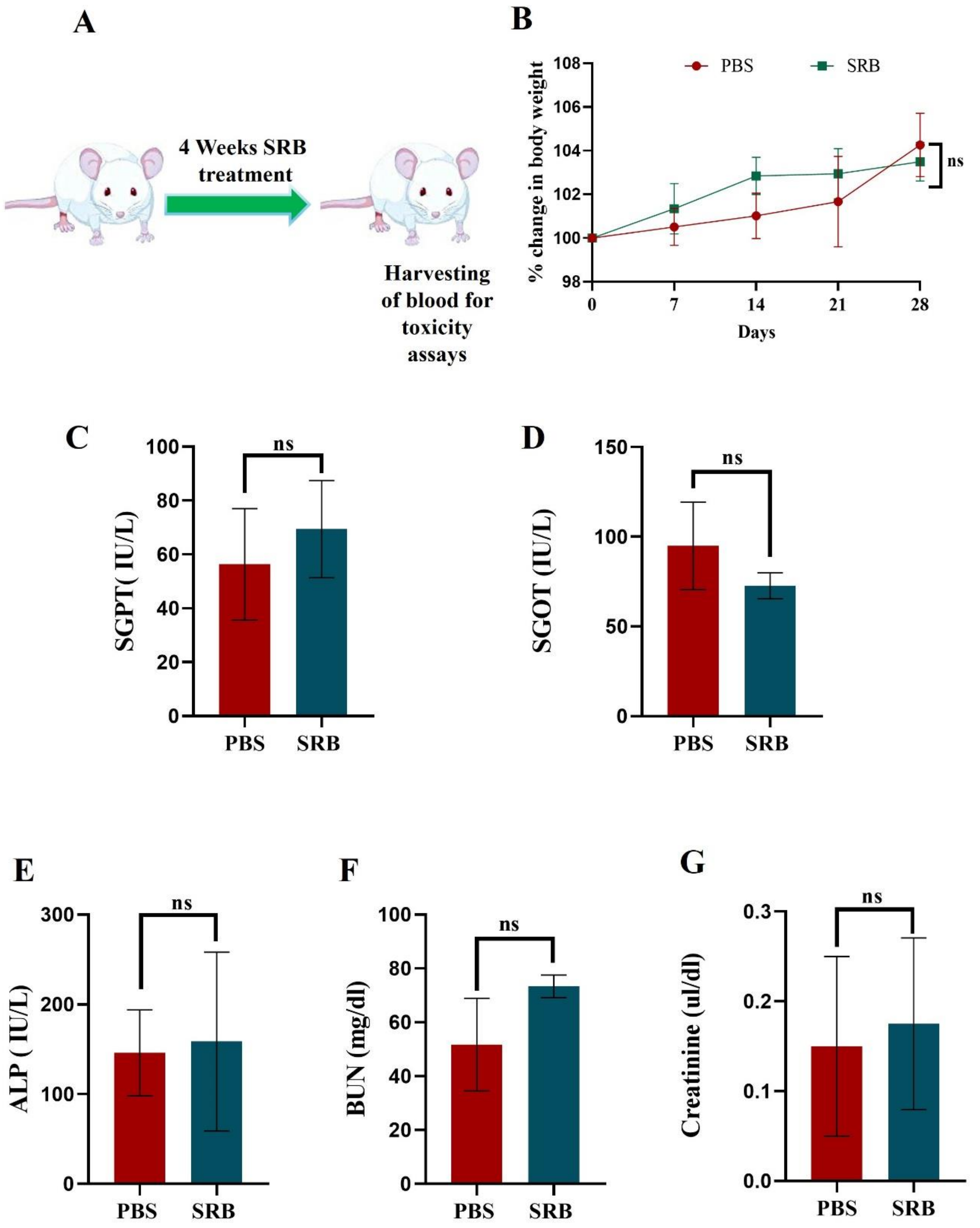

**Supplementary Figure 2. SRB shows no toxicity in mice. (A)** Schematic of SRB drug treatment in BALB/C mice for toxicity examination**. (B)** Percentage of weight gain and loss in SRB-treated and PBS control mice (n=5) of toxicity experiment. Comparative values of SGPT **(C),** SGOT **(D)**, ALP **(E)**, BUN **(F)** and creatinine **(G)** of the SRB treated and PBS control groups, showing non-significant(ns) changes. These parameters indicate that the SRB shows no significant hepatotoxic and nephrotoxic effects. Data information: data are obtained from 4 biological replicates and presented as mean ± SD and the statistical significance between experimental groups was determined by an unpaired Student's t-test (*P < 0.05, **P < 0.01, ***P < 0.001, ****P < 0.0001, and n.s. not significant.

## Supplementary Figure 3

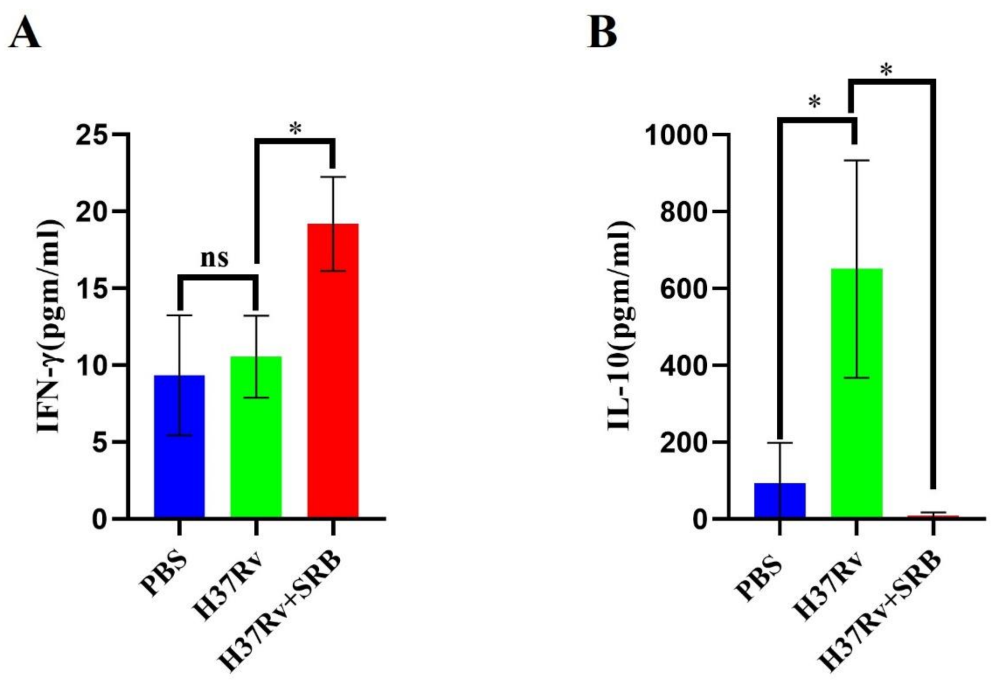


**Supplementary Figure 2.** For the protein level assessment of cytokines in supernatant tissue homogenates, we have used the BD™ CBA Mouse Th1/Th2/Th17 Cytokine Kit (Catalog No. 560485) to Interferon-gamma (IFN-γ), and Interleukin-10 (IL-10) protein levels**(A&B),** respectively. Data information: data are obtained from 3 biological replicates and presented as mean ± SD and the statistical significance between experimental groups was determined by an unpaired Student's t-test (*P < 0.05, **P < 0.01, ***P < 0.001, ****P < 0.0001, and n.s. not significant
